# Buried bumper syndrome in percutaneous endoscopic gastrostomy with a jejunal extension tube in patients undergoing levodopa–carbidopa intestinal gel treatment

**DOI:** 10.1186/s40792-023-01785-7

**Published:** 2023-12-11

**Authors:** Masatoshi Nakagawa, Noboru Inoue, Shuhei Takise, Masashi Takayanagi, Tsukasa Kubo, Hiroto Muroi, Masanobu Nakajima, Shinji Morita, Takatoshi Nakamura, Kazuyuki Kojima

**Affiliations:** 1 Department of Upper Gastrointestinal Surgery, Dokkyo Medical University, 880 Kitakobayashi, Mibu-Machi, Shimotsuga-Gun, Tochigi 321-0293 Japan; 2 Department of Surgical Oncology, Dokkyo Medical University Graduate School of Medicine, 880 Kitakobayashi, Mibu-Machi, Shimotsuga-Gun, Tochigi 321-0293 Japan

**Keywords:** Buried bumper syndrome, Percutaneous endoscopic gastrostomy with a jejunal extension tube, Levodopa–carbidopa intestinal gel

## Abstract

**Background:**

Levodopa–carbidopa intestinal gel (LCIG) treatment is an effective Parkinson’s disease (PD) treatment that requires percutaneous endoscopic gastrostomy with a jejunal extension tube (PEG-J). Buried bumper syndrome (BBS) is an uncommon but significant complication of PEG-J for LCIG.

Case presentation

A 71-year-old man had been undergoing LCIG therapy for PD since a PEG-J was implemented at our department two years previously. He presented with appetite loss. Computed tomography showed that the gastrostomy bumper was buried in the gastric wall. The patient was surgically treated with the simultaneous removal and replacement of PEG-J. Postoperative gastrocutaneous fistula occurred, which was conservatively treated.

**Conclusions:**

Notably, patients and medical staff should be aware that patients with PD on LCIG treatment have a high risk of BBS in PEG-J and that there might be some patients with latent BBS. When simultaneous removal and replacement surgery is performed, establishing a new route at the stomach and abdominal wall is recommended.

## Background

Levodopa–carbidopa intestinal gel (LCIG) treatment is effective for advanced Parkinson’s disease (PD). LCIG is continuously delivered to the upper intestine, ensuring stable levodopa plasma levels compared with standard oral levodopa therapy. This reduces motor response fluctuations and improves non-motor complaints commonly associated with chronic oral levodopa treatment [1–3]. Percutaneous endoscopic gastrostomy with a jejunal extension tube (PEG-J) is needed to perform LCIG treatment.

Buried bumper syndrome (BBS) is a rare complication after PEG tube placement with an approximate frequency of 0.3–2.4% [4]. BBS may present as increased leakage around the PEG tube, resistance to infusion, or abdominal pain with an infusion of feed and can be confirmed clinically by an inability to advance and rotate the tube [4, 5]. Patients with PD under LCIG treatment can usually eat orally and do not need enteral feeding using a PEG-J gastric tube. However, they require continuous LCIG administration through a jejunal tube of PEG-J. Therefore, they tend to need PEG-J less frequently than patients needing enteral feeding through a PEG tube. Thus, BBS might be unnoticed until severe symptoms occur or are incidentally identified. It was reported that the frequency of BBS in PEG-J for PD on LCIG treatment was 7.4–17.1%, which was much higher than that of BBS in PEG in general [6, 7]. The median interval time between placement of PEG-J and occurrence of BBS was reported to be 25.5 months [6].Once BBS in PEG-J occurs, simultaneous removal and replacement are ideal to avoid LCIG treatment cessation, but detailed information for its surgical procedure is yet to be introduced.

Here, we present a case of BBS in PEG-J in a patient who had underwent LCIG therapy. We surgically treated the patient to remove the PEG-J and simultaneously replaced it with another one.

## Case presentation

### Details of present illness and medical history

A 71-year-old man presented with appetite loss at the internal medicine department of our institute. Computed tomography (CT) showed a splenic abscess requiring intravenous and oral antibiotics therapy for 3 months. A follow-up CT revealed splenic abscess disappearance, but the gastrostomy bumper was buried in the gastric wall. He was referred to our department for further examination and treatment. His medical history included PD and cerebral infarction without sequelae. He had been undergoing LCIG therapy for 2 years since a PEG-J was implemented at our department.

### Preoperative examination and imaging findings

**Fig. 1:**
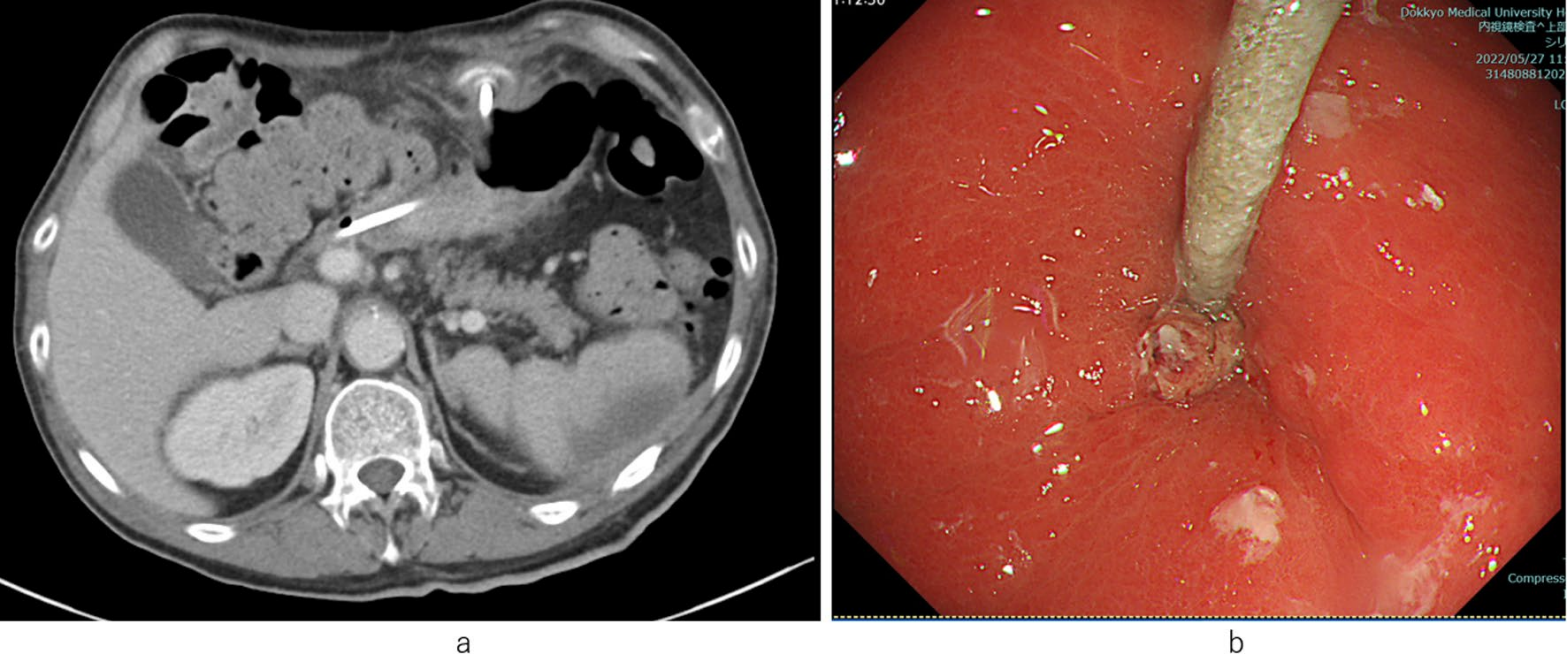
Preoperative imaging findings. **a** CT revealed an internal bumper of PEG-J buried in the gastric wall. **b** EGD showed a complete BBS of PEG-J. PEG-J, percutaneous endoscopic gastrostomy with a jejunal extension tube; BBS, buried bumper syndrome; *EGD* esophagogastroduodenoscopy; *CT* computed tomography

Abnormal laboratory test findings at the first visit to our outpatient clinic included levels of hemoglobin, 12.5 g/dL; urea nitrogen, 27.0 mg/dL; and CRP, 0.39 mg/dL. CT showed BBS in PEG-J in the gastric wall (Fig. 1a), which was confirmed as complete BBS on esophagogastroduodenoscopy (EGD) (Fig. 1b). Simultaneous removal and replacement of PEG-J was planned.

### Surgery and clinical course

**Fig. 2:**
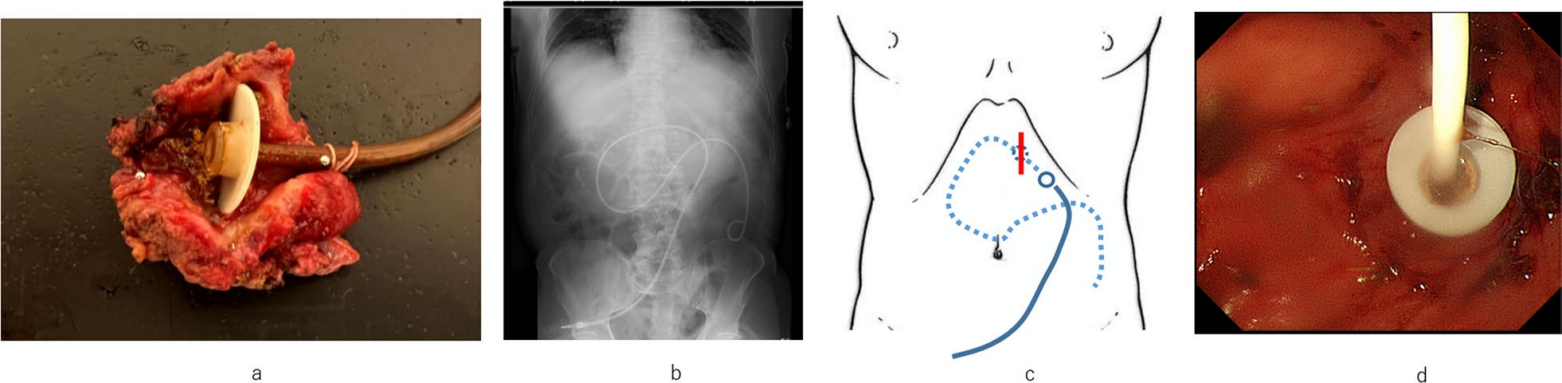
Surgical findings.** a** The internal bumper was buried entirely in a resected fistula.** b** Intraoperative fluoroscopy confirmed new PEG-J placed in the proximal jejunum. **c** A surgical schema showing skin incision and PEG-J site. **d** Intraoperative EGD confirming the internal bumper inside the stomach and no gap around the tube. PEG-J, percutaneous endoscopic gastrostomy with a jejunal extension tube; *EGD* esophagogastroduodenoscopy

At first, a guidewire was inserted into the jejunum through a preexisting PEG-J, confirmed with intraoperative fluorography. A spindle-shaped skin incision was made around the PEG-J. Laparotomy was performed by removing the fistula. The PEG-J was removed with leaving the guidewire. Resection margin 5 to 10 mm was secured so that remnant gastric wall remained intact (Fig. 2a). A new PEG-J was placed in the jejunum along the guidewire (Fig. 2b and c). As the stomach defect was larger than the size of PEG-J, interrupted single whole-layer suture was used to close it. Intraoperative EGD was performed to confirm no gap between the PEG-J and gastric wall around it (Fig. 2d). The PEG-J was then exteriorized through the different parts from the previous wound (lateral and caudal). The abdomen was then closed, and the operation was completed. Operative time was 2 h 43 min, and intraoperative blood loss was 49 mL.

Pathological findings revealed erosion and ulceration at the mucosal layer, and inflammatory granuloma tissue and abscess were found. There was congestion and bleeding in most layers of the gastric wall. These findings suggested circulation impairment, which is consistent with BBS.

**Fig. 3:**
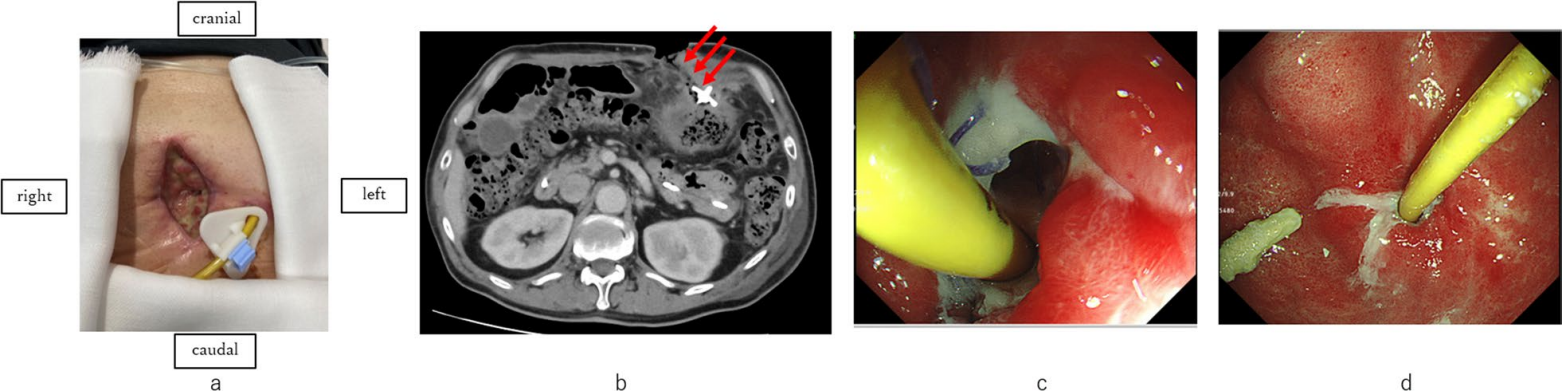
Postoperative findings. **a** Wound dehiscence occurred, and food residue exuded from a gastrocutaneous fistula. **b** CT showing a gastrocutaneous fistula (red arrows). There was no sign of free air or ascites. **c** EGD revealed a gap around the PEG-J. **d** A follow-up EGD showed the disappearance of the gap around the PEG-J.PEG-J, percutaneous endoscopic gastrostomy with a jejunal extension tube; *EGD* esophagogastroduodenoscopy; *CT* computed tomography

LCIG therapy was continued just before the operation since jejunal tube was patent. Intravenous administration of levodopa was performed in the evening on the operation day and in the morning on postoperative day (POD) 1. LCIG therapy was reinitiated in the afternoon on postoperative day 1. On POD 2, oral water intake was initiated. Wound redness occurred, and wound irrigation was started. White blood cell count and CRP levels were elevated to 14000/µL and 26 mg/dL, respectively. A soft diet was initiated on POD 3. Wound dehiscence occurred, and negative wound pressure therapy was started on POD 5. On POD 9, food residues leaked out of the wound (Fig. 3a). Emergent CT showed a fistula between the wound and the gastric wall where PEG-J was inserted (Fig. 3b). EGD revealed a gap around PEG-J (Fig. 3c). As the CT showed no sign of peritonitis, such as free air or ascites around the PEG-J, the patient received no oral treatment, total parenteral nutrition, nasogastric tube drainage, and antibiotics. The patient did not suffer from fever or elevation of white blood cell count or CRP after the event. On POD 12, food residue stopped exuding from the wound, and EGD on POD 27 showed the disappearance of the gap around the PEG-J (Fig. 3d). On POD 28, soft diet was reinitiated. Following this, the clinical course was uneventful, and the patient was discharged on POD 38.

## Discussion

We encountered a rare case of BBS in PEG-J in a patient who had undergone LCIG, which was surgically treated with simultaneous removal and replacement. The patient suffered from a postoperative gastrocutaneous fistula, which recovered conservatively.

Excessive compression of tissue between the external and internal fixation device of the gastrostomy tube is considered the main cause of BBS. Factors related to development of BBS include the characteristics of internal bumper (Freka type), malnutrition, increase in abdominal wall thickness [8], and excessive external traction on the tube causing friction and ischemic necrosis of the gastric submucosa [9]. Local infection causes erythema, purulent secretion and pain. Other symptoms include inability to insert, loss of patency, and leakage around the PEG tube.

Various treatment methods for BBS in PEG have been introduced [8–13]. Endoscopic treatment is an option, as it is less invasive than surgical intervention [9, 11]. However, complicated or difficult cases should be treated with surgery [12, 13]. BBS in PEG-J has several characteristics different from those of BBS in PEG. If removal and replacement cannot be achieved simultaneously, the treatment should be divided into two stages; removal and replacement. Therefore, temporal alternative treatment for PD should be performed by administering oral, intravenous, or nasojejunal medication.

Following are some recommendations for surgery involving simultaneous removal and replacement. In the present case, to easily insert a new jejunal tube into the jejunum, a guidewire was inserted into the previous PEG-J, ensuring the route to the jejunum was confirmed using intraoperative fluoroscopy. The new PEG-J was inserted at the same site in the gastric wall and a different site in the abdominal wall. The gap between PEG-J and the gastric wall was closed with a suture, and the gastric wall was fixed to the abdominal wall as usual; however, a postoperative gastrocutaneous fistula occurred. Inflammation of BBS was observed, and the gastric wall was friable, which caused a laceration of the gastric wall leading to a gastrocutaneous fistula. This should have been managed by closing the previous fistula in the gastric wall and inserting a new PEG-J through a different site. In cases where a different route to the gastric wall is difficult to make, the procedure should be divided into two stages to ensure safety. Surgical or endoscopic removal of the PEG-J is performed first. After wound inflammation stabilizes, a new one is inserted at the same site.

Patients with PD undergoing LCIG treatment have an increased risk of BBS than those without PD [6]. As patients with PD patients undergoing the LCIG therapy can eat orally, they do not require gastric tubes of PEG-J, making PEG-J care less frequent. If a small caliber of the jejunal tube remains patent and functional, a delay in diagnosing BBS can occur until it is noticed incidentally or a symptom appears [13]. Patients with PD tend to gain weight if LCIG treatment is effective, increasing the width of abdominal wall. An internal bumper of PEG-J for LCIG is designed as a Freka type, which has a higher risk of BBS than other types of bumper [14]. As mentioned above, patients with PD undergoing LCIG treatment have an increased risk of BBS, which often goes unnoticed by the patients, their families, and even medical staff, posing a serious problem. Regular monitoring of the PEG-J with daily water flushes and ensuring the distance between skin and external bumper to be at least 1 cm and tube mobility are recommended to prevent BBS [15].

## Conclusions

In conclusion, there is a need for increased awareness among patients and medical staff regarding the high risks of BBS in patients with PD undergoing LCIG treatment and the possibility of some patients having latent BBS. When simultaneous removal and replacement surgery is performed, establishing a new route at the stomach and abdominal wall is recommended.

## Data Availability

Not applicable.

## Abbreviations

LCIG: Levodopa–carbidopa intestinal gel
PD: Parkinson’s disease
PEG-J: Percutaneous endoscopic gastrostomy with a jejunal extension tube
BBS: Buried bumper syndrome
CT: Computed tomography
EGD: Esophagogastroduodenoscopy
POD: Postoperative day
